# Assessment of Oral Hygiene Practices and Associated Risk Factors Among Dental Patients in Kabul, Afghanistan: A Cross-Sectional Study

**DOI:** 10.1155/ijod/7840384

**Published:** 2025-09-18

**Authors:** Ali Maisam Eshraqi, Abdurrahman Anwari, Sayed Esa Sadaat, Mohammad Haris Taheri, Arash Nemat, Elaha Sumaya Ghafari, Ahmad Siyar Noormal

**Affiliations:** ^1^Department of Endodontics and Operative Dentistry, Kabul University of Medical Sciences “Abu Ali Ibn Sina”, Kabul, Afghanistan; ^2^Department of Social Statistics and Demography, University of Southampton, Southampton, UK; ^3^Department of Global Public Health, Karolinska Institutet, Stockholm, Sweden; ^4^Department of Periodontics, Kabul University of Medical Sciences “Abu Ali Ibn Sina”, Kabul, Afghanistan; ^5^Department of Epidemiology of Transition, Heidelberg Institute of Global Health, Heidelberg, Germany

**Keywords:** Afghanistan, education, oral hygiene, snacks, socioeconomic factors

## Abstract

**Background:** Oral health is a critical aspect of overall well-being, with significant implications for physical health and quality of life. Despite the well-documented understanding of oral hygiene practices, there is limited research on oral health behaviors in Afghanistan, a country with unique sociocultural and economic challenges.

**Objectives:** This study aims to assess oral hygiene practices and identify associated risk factors among individuals in Kabul, Afghanistan, to provide insights into oral health behaviors in low-resource settings.

**Methods:** Institutional based cross-sectional study was conducted from March 2021 to March 2022, involving 1948 participants from dental hospitals in Kabul. Descriptive and inferential statistical analyses, including logistic regression, were employed to explore associations between variables and oral hygiene status.

**Results:** The study revealed that 59.7% of participants had poor oral hygiene. Key predictors of good oral hygiene included higher education levels, better wealth status, and younger age. Surprisingly, higher snack consumption was associated (AOR: 1.66, CI: 1.24–2.21) with better oral hygiene, contrary to existing literature. Continuous medication use also correlated with better oral hygiene practices with an AOR of 2.14.

**Conclusions:** The findings highlight the complex interplay of demographic, educational, and behavioral factors in determining oral hygiene practices. Targeted interventions including health information provision regarding oral health for patients specifically for poor and uneducated individuals is recommended to enhance oral health outcomes in low-resource settings like Afghanistan.

## 1. Introduction

Oral health is an integral component of overall well-being, which influences not only individual's physical health but also their psychosocial and emotional aspects of life. According to the Federal Dental International (FDI), oral health encompasses a spectrum of abilities, including chewing, tasting, touching, smelling, swallowing, smiling, speaking, and conveying most of emotions through facial expression without any discomfort, pain, and disease of the craniofacial region [1].

Over the past three decades, global prevalence rates of periodontal diseases, dental caries, and oral cancers have increased by an average of 45.6%, which reinforces the need for a closer examination of oral health practices [2]. Importantly, poor oral hygiene behaviors, leading to dental infections, emerged as an independent risk factor for various chronic systemic diseases, including cardiovascular diseases, diabetes, stroke, digestive diseases, adverse pregnancy outcomes, and obesity [3–7]. Conversely, it is essential to recognize that systemic disorders may also contribute to the emergence of oral diseases [8, 9].

Maintaining optimal oral hygiene and preventing conditions such as dental caries and periodontal diseases͵ necessitates adherence to recommended oral care regimen, including brushing teeth at least twice daily and utilizing complementary tools such as dental floss and interproximal brushes [10, 11].

Despite this well-documented understanding, oral health remains underemphasized globally with studies revealing significant variation in adherence to a recommended oral hygiene practices across different income countries and cultural contexts [2]. Notably, higher rates of unhealthy oral hygiene practices are observed in low and middle-income countries like India (52.2%), Lebanon (35%), and Turkey (32%), compared to higher rates in high-income countries like Italy (7.9%) and the USA (25%) [6, 12–15]. This variation in adherence to oral hygiene practices underscores the multifaceted nature of oral health behaviors influenced by socioeconomic factors and cultural norms.

Afghanistan for several years, suffered from social inequity, poverty, and ongoing conflicts, which have profoundly affected not only the population's general well-being, but also their oral health and nutrition [16, 17]. Despite many efforts to promote health in Afghanistan, oral health has received limited attention, and the research on oral hygiene practices and associated risk factors in Afghanistan is limited. There are very few available evidence in Afghanistan, that assess the oral hygiene status. A study that was conducted among children in Kabul National Stomatology hospital showed that majority of children in their study had a fair oral hygiene status [18], while another study conducted among university students at the Kabul University of Medical Sciences (KUMS) “Abu Ali Ibn Sina” exhibited that around 44% of participants were brushing their teeth twice per day [19].

Additionally, some targeted efforts have been made in Afghanistan to improve oral health and raise awareness, particularly among children in Kabul city. Some key initiatives, supported by the World Health Organization and the Ministry of Public Health were launched in the past which focused on training of school teachers in oral health practices, however, there are few recent efforts in this capacity. The Afghanistan Dental Relief Project (ADRP) is another initiative which plays a role in promoting oral health in Afghanistan. In 2019, it partnered with some Kabul city schools to deliver oral health education, dental checkups, and hygiene kits to students in underserved areas. ADRP [20] also works with maternity hospitals to educate pregnant women on oral care for themselves and their newborns. However, the impact of these efforts has yet to be systematically evaluated or publicly reported.

Given the scarcity of reliable data and the unique socio-cultural and infrastructural context of Afghanistan, it is essential to produce high-quality evidence on oral health behaviors to inform effective public health interventions. Most existing studies have focused on specific subgroups, such as children and students, with little information available on the general population.

Considering all the above-mentioned points and the lack of existing literature on oral hygiene in Kabul, Afghanistan, this study is being conducted to address this knowledge gap and contribute scientifically to inform relevant public health efforts in Afghanistan. The primary objective of this study is to assess oral hygiene practices and associated risk factors among individuals in Kabul, Afghanistan. Through a comprehensive exploration of oral health behaviors, the study aims to contribute to the existing body of knowledge on oral health in low-resource settings and guide evidence-based interventions.

## 2. Methodology

### 2.1. Study Design

This quantitative cross-sectional study was conducted within Kabul city from March 2021 to March 2022. Kabul, the capital and largest city of Afghanistan, consists of 22 districts and is central to Kabul Province. With an estimated population of approximately 3 million people in 2020 [21], Kabul hosts primarily two main public dental hospitals and several private dental clinics for dental services.

### 2.2. Sampling Technique and Sample Size

Utilizing a convenience sampling method, participants visiting dental hospitals and clinics were selected. Sample size determination followed the single population proportion formula, assuming a 50% prevalence of poor oral hygiene practice, with a 95% confidence interval, 2.5% margin of error, and 5% nonresponse rate. The initial calculated sample size was 1537, later increased to 2000 considering the poor oral hygiene situation and design effect of the sampling strategy. Ultimately, data from 1948 participants were collected, including all patients attending the specified healthcare institutions during the survey period, except for those with emergency conditions or those who declined participation. As we have used convenience sampling approach, we acknowledge that the study population may not fully represent all individuals in Kabul, particularly those who do not seek dental care or visit health facilities.

### 2.3. Data Collection

Data collection primarily occurred at the Stomatology Teaching Hospital, Afghanistan's largest national dentistry hospital and at one randomly selected private dental clinic. A structured interviewer-administered questionnaire, available in Persian and Pashtu, was utilized for face-to-face interviews conducted after patients received treatment. The questionnaire was adapted from an existing instrument used at the Stomatology Teaching Hospital and modified based on the authors' expertise to align with the study objectives and the local context. To our knowledge, no validated tool was available for this setting at the time of data collection, which we acknowledge as a limitation of the study. The questionnaire was pretested with 50 individuals to ensure clarity, relevance, and cultural appropriateness. Six first- and second-year trainees/residents were trained to collect data, and supervised by two professional dentists. As the data collection relied on self-reported responses, there is a potential for information bias, including recall bias and social desirability bias, which may have led participants to over-report positive hygiene practices or under-report unhealthy behaviors.

### 2.4. Measurement

The questionnaire comprised sections on socio-demographic characteristics, oral hygiene practices, health behavior, and potential oral health risk factors.

### 2.5. Data Quality Control

A pretest of the questionnaire was conducted among 50 individuals, and necessary amendments were made based on the results. Data collectors and supervisors underwent comprehensive training on study objectives and procedures. These measures were implemented to reduce potential information bias by improving the clarity of the questionnaire and ensuring consistency in how questions were asked and recorded during interviews. The finalized questionnaire was used for data collection, and the entered data were systematically coded, exported, cleaned, and analyzed using STATA software version 12. Double checks were performed during data entry, and a final random cross-check was conducted at the dental facility.

### 2.6. Analysis

The primary outcome of this analysis was oral hygiene practices, treated as a binary outcome. Potential explanatory variables included sex, age, wealth, education, beverage consumption, sweets and citric fruits consumption, gastric diseases, bruxism, xerostomia, and continuous medication usage. The outcome variable was created based on participants' responses to three specific questions regarding teeth-brushing habits, dental floss usage, and regular oral check-ups. Participants were classified as having “good oral hygiene” if they engaged in at least one of the following practices: brushing their teeth at least twice a day, regular use of dental floss, or undergoing oral health check-ups annually. It is important to note that our operational definition of “good oral hygiene” reflects a minimal threshold suited to the context of Afghanistan. Currently, there is no universally accepted threshold for defining “good oral hygiene” in population-based studies. Definitions vary widely depending on study aims, available data, and local context. For example, a community-based survey in rural Ethiopia categorized households as having “good oral hygiene” based on simple behaviors like brushing, mouth rinsing after meals, and avoidance of harmful practices [22]. Similarly, an adapted oral hygiene behavior index emphasizing culturally appropriate behaviors such as brushing and tongue cleaning used in Nepal [23]. This pragmatic definition allows for meaningful interpretation of oral hygiene behaviors in settings such as Afghanistan, where access to dental care and oral health education may be limited.

Descriptive analysis, comprising simple frequencies and measures of central tendency, was utilized to characterize the sample using summary measures and tables. Chi-square tests and binary logistic regression were employed to explore associations between independent variables and the outcome variable. Finally, we performed multiple logistic regression incorporating all variables that showed significant association in the bivariate analysis. All variables that showed statistically significant associations in the bivariate analysis (*p* < 0.05) were considered potential confounders and were included in the multivariable logistic regression model to control for their effects and obtain adjusted odds ratios (AORs). The level of statistical significance was established at a *p*-value <0.05. Multicollinearity was assessed using variance inflation factors (VIFs), and model fit was evaluated using the Hosmer–Lemeshow goodness-of-fit test.

### 2.7. Ethical Considerations

The survey protocol received official approval (letter number 158) from the Institutional Review Board of KUMS. Participants provided consent forms before participation, with minors requiring consent from parents or guardians. Throughout the study, efforts were made to uphold patient privacy and data confidentiality.

## 3. Results

**Figure 1:**
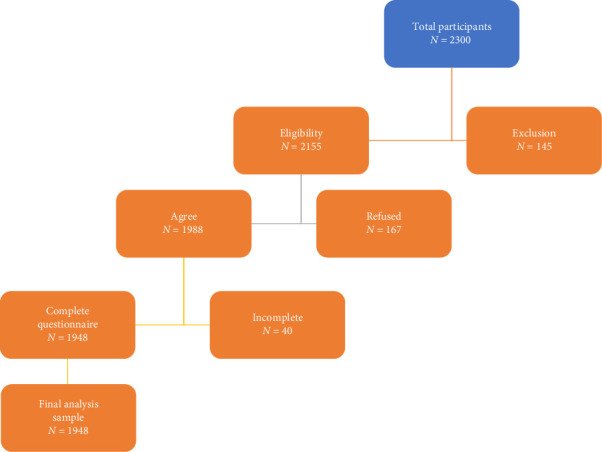
Flowchart of participants' selection.

Out of 2300 individuals initially approached for participation, 2155 met the eligibility criteria. Among those eligible, 1988 agreed to participate, with 1948 completing the questionnaire fully.  Figure 1 shows the detailed flow of participants through the study stages.

The socio-demographic characteristics of participants are shown in Table 1.

Among the 1948 participants, 43.1% were males and the majority were in the age group of 18–34 years old. The mean age of the study population was 28.6. Regarding the wealth status, approximately 63% were in the middle class, 22.8% had good wealth, and 13.4% were poor. Education-wise, over 44% were high school graduates, 23% had higher education, and 17.1% were illiterate. Almost two-thirds reported consuming beverages “sometimes,” while approximately 20% did not consume beverages. Similarly, two-thirds (58.1%) of the participants reported consuming sweets and candy “sometimes,” with 15.5% reporting never consumption. Additionally, 44% reported consuming snacks “sometimes,” while 16.1% reported usually consuming snacks.

A majority of participants did not report gastric diseases (68.7%), xerostomia (79.7%), and bruxism (81.1%); however, nearly 15% reported using medication continuously.

Dental floss usage was low, with around 48% not using it at all and only 11% reporting “usual” usage. Furthermore, 31% reported brushing their teeth before sleeping, 29% in the morning, while around 10% did not brush their teeth at all.

The distribution of dental check-ups was notable. Over half of the participants sought dental care only when experiencing a toothache, with 34% not undergoing regular check-ups. Meanwhile, 7.73% and 6.6% visited the dental office every 6 months and yearly, respectively.

There were some missing values for individual variables; however, participants with missing data on any of the variables included in the regression analysis were excluded using listwise deletion. As a result, the final multivariable logistic regression model was performed on 1848 participants with complete data on all variables.

Table 2 presents the distribution of oral hygiene practices across various demographic and behavioral categories, highlighting notable trends. The overall prevalence of poor hygiene was higher among the study participants standing at almost 60% compared to 40% of good hygiene practices. In terms of sex, it was almost evenly distributed in both groups. Additionally, age appeared to be a significant factor, as a larger percentage of individuals aged 45 or above demonstrated poor oral hygiene compared to those below 45.

Wealth status also influenced oral hygiene practices, with individuals in the “good” wealth category exhibiting better oral hygiene practices compared to those in the “middle” and “poor” categories. Moreover, educational attainment demonstrated a similar trend, with higher education levels correlating with better oral hygiene practices.

Behavioral factors such as snack consumption, sweet and candy consumption, and continuous medication usage also demonstrated associations with oral hygiene practices, with varying proportions of good and poor oral hygiene across different levels of these behaviors. These findings underscore the multifaceted nature of oral hygiene practices and highlight the importance of considering various demographic and behavioral factors in promoting oral health awareness and practices within the population.

**Figure 2:**
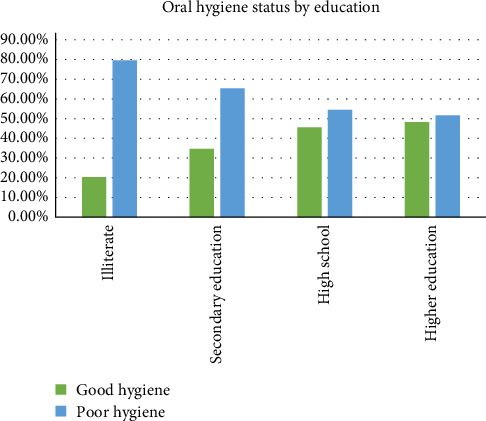
Oral hygiene status of the participants according to their education attainment.

**Figure 3:**
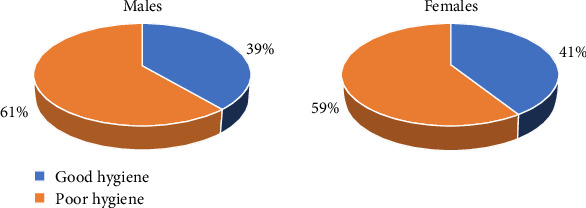
Oral hygiene status of the participants by gender.

Figure 2 presents the oral hygiene status of the participants according to their education level. As can be seen in the chart, the percentage of individuals with good oral hygiene increases with higher education attainment. Participants in the lowest education category represent the highest proportion of poor oral hygiene, while those with higher education levels represent better oral hygiene practices. Moreover, Figure 3 shows the distribution of oral hygiene status by gender. The figure shows that oral hygiene practices are almost similarly distributed between men and women.

The logistic regression analysis (Table 3) revealed several significant predictors of oral hygiene practices among the study population. Prior to interpreting the regression findings, we assessed model assumptions and diagnostics. Multicollinearity among independent variables was evaluated using VIFs, with all values below 5, indicating no significant multicollinearity. The Hosmer–Lemeshow goodness-of-fit test yielded a chi-square of 9.39 (df = 8; *p*=0.31), indicating an adequate fit of the logistic regression model to the data.

Age emerged as a notable factor, with individuals above the age of 45 exhibiting significantly lower odds of good oral hygiene compared to their younger counterparts, even after adjusting for other variables. Education level demonstrated a strong association with oral hygiene, as higher levels of education were consistently associated with better oral hygiene practices. Wealth status showed a significant association in the crude analysis, with individuals in the middle and good wealth categories having higher odds of good oral hygiene. After adjusting for other variables, the association with middle category did not remain significant; however, those in the good wealth status showed higher likelihood of having good hygiene after adjustment. Interestingly snack consumption shows significant association with oral hygiene status, with individuals who reported consuming snacks having higher odds of good oral hygiene compared to nonconsumers. Additionally, continuous medication use was associated with higher odds of good oral hygiene, indicating a potential link between medication regimen and oral health behaviors. Surprisingly, sex did not significantly predict oral hygiene practices after adjusting for other variables. These findings underscore the multifactorial nature of oral hygiene practices, highlighting the importance of age, education, snack consumption, and medication use in shaping individual oral health behaviors.

## 4. Discussion

This study aimed to assess oral hygiene practices and identify the factors that influence them among people in Kabul, Afghanistan, and the results provide important insights into these aspects. We found that 59.7% of participants had poor oral hygiene, highlighting the ongoing challenges to oral health in these low-resource areas.

The findings of this study largely reflect the oral hygiene habits of a predominantly young adult population in Kabul. Many participants were from middle or higher income groups and had at least a high school education. Because of this, the results may not fully generalize to older adults, people with lower incomes, or those living in rural areas or different cultural settings. Oral hygiene behaviors such as brushing, flossing, and snack consumption are often shaped by local resources, traditions, and education levels. While this research provides valuable insight into oral health practices in an urban, low-resource environment, caution is needed when extending the findings to populations with different characteristics. More studies in diverse settings are essential to better understand the wider relevance of these results.

The analysis identified several predictors of good oral hygiene, including higher education, greater wealth, and younger age. These factors reflect broader social and economic influences on health behaviors. One surprising result was the positive link between higher snack consumption and better oral hygiene practice, which suggests that unique local circumstances may be shaping this relationship. Another notable finding was the association between continuous medication use and improved oral hygiene, pointing to possible opportunities for targeted health interventions.

Overall, these findings highlight both the prevalence of oral hygiene issues and the complex ways demographic factors, daily habits, and social conditions interact to influence oral health in Kabul, Afghanistan. By identifying key predictors, this study offers valuable guidance for designing oral health strategies that are informed by the specific social and economic realities of the population.

Age emerged as a key determinant of oral hygiene practice, with individuals aged 45 and above demonstrating significantly lower odds of good oral hygiene. This finding, which is in line with regional and global trends [24–27], underscores the importance of age-related factors in shaping oral health behaviors, suggesting a potential need for targeted interventions tailored to older age cohorts to improve oral hygiene practices and mitigate oral health risks.

Education level demonstrated a robust association with oral hygiene, with higher educational attainment consistently linked to better oral hygiene practices. This finding aligns with existing literature [27–29] highlighting the role of education as a determinant of health behaviors, including oral hygiene practices. Higher levels of education may confer greater awareness of the importance of oral health and access to resources for maintaining optimal oral hygiene, thereby influencing individual behaviors and practices.

Higher socioeconomic status was found to be linked to improved oral hygiene practices, highlighting a relationship between wealth and better oral health habits. Individuals categorized under the “good wealth status” are more likely to exhibit positive oral hygiene practices. This is in line with the existing literature. A systematic review which was conducted in 2020, demonstrates a direct correlation between socioeconomic status and oral health-related quality of life, indicating that individuals with lower socioeconomic statuses tend to experience poorer oral health outcomes [30]. Those in the good wealth category typically have better access to oral health education, preventive dental care, and resources such as fluoridated toothpaste and regular dental check-ups. Conversely, individuals in middle and lower wealth statuses often face barriers such as limited access to affordable dental services and lower awareness of effective oral hygiene practices. These findings underscore the importance of targeted interventions aimed at improving oral health behaviors among populations in middle and lower wealth categories, thereby mitigating disparities and promoting better overall oral health outcomes.

Surprisingly, our study revealed a noteworthy positive association between snack consumption and oral hygiene status. This finding is contradicting some existing literature which suggests negative association between oral hygiene status and frequent snack consumption [31, 32]. Furthermore, studies in South and East Asian countries have shown that the occurrence of dental caries is significantly higher among children who frequently consume sugary snacks [33–35]. This finding may be explained by several factors. First, the types of snacks commonly used in Afghanistan differ from those in other contexts. Usually, Afghans consume some types of healthy foods as snacks which are less sugary which may not have a detrimental impact on oral health. Second, higher snack consumption might be indicative of a good wealth status, which would be associated with better oral health practices and behaviors. This finding emphasizes the importance of considering factors like the frequency of snacking and oral hygiene practices postconsumption in understanding their impact on oral hygiene practices. This highlights the need for a more comprehensive approach to examining how dietary habits, including snacking behaviors, influence overall oral hygiene.

The observation that continuous medication use correlates with higher odds of maintaining good oral hygiene suggests a potential connection between medication routines and oral health practices. This finding implies that individuals who adhere to ongoing medication regimens may benefit from regular interactions with healthcare providers, potentially leading to better education and awareness regarding oral hygiene. The exposure to healthcare settings and ongoing medical care may contribute to improved oral health behaviors, highlighting the broader impact of healthcare access and education on overall oral hygiene practices. Thus, the relationship between medication use and oral hygiene underscores the importance of integrated healthcare approaches in promoting comprehensive health outcomes. It is important to acknowledge that our definition of “good oral hygiene” reflects a minimal behavioral threshold appropriate to the Afghanistan context. While there is no universal standard for defining “good oral hygiene” in epidemiological studies, we used a context-appropriate behavioral threshold based on the presence of at least one preventive practice. We acknowledge that this definition may differ from more comprehensive criteria used in clinical or high-resource settings, which should be considered when comparing results across different populations.

The finding that sex did not predict oral hygiene practices after accounting for other variables is not surprising, as gender itself does not inherently dictate oral health behaviors. Instead, it is proposed that individuals' attitudes and behaviors towards health are mostly shaped by life experiences and events. Although some studies have found that women are more likely to maintain good oral hygiene practices, in our study we did not find any difference [36]. Factors such as upbringing environment, education, and experiences throughout childhood play key roles in shaping perceptions and practices related to health [37]. The availability of education for men and women in Afghanistan has potentially contributed to equal oral hygiene practices between the two genders. The effect may have been either through direct education or through influence from other family members and society at large.

Considering the results of our research, public health programs in Kabul should focus on improving oral hygiene education, especially for older adults and individuals with lower levels of education and income, as these groups showed poorer oral health practices. Effective approaches may include community workshops, school-based programs, and improving the affordability and accessibility of dental care to encourage regular brushing, flossing, and dental check-ups.

Future studies should use longitudinal designs to track how changes in behavior, education, and income influence oral health over time. Trials that provide free dental hygiene supplies or mobile dental services could also help identify effective ways to improve oral health in low-resource settings. Such efforts are essential for addressing existing gaps and achieving sustainable improvements in oral hygiene in Kabul and similar communities.

### 4.1. Strengths and Limitation

The large sample size of 1948 participants, which enhances the reliability and statistical power of analysis, is a key strength of this study. Using a structured, interviewer-administered questionnaire for detailed data collection across diverse demographics is a plus point for this study. Importantly, this research represents a pioneering effort in examining oral health behaviors in Afghanistan, a context with limited existing research. However, the study also has notable limitation. The cross-sectional design restricts causal inferences. Additionally, the convenience sampling strategy might have introduced selection bias, as participants were drawn from specific dental hospitals and clinics. This approach may limit the representativeness of the findings and restrict their generalizability to the broader population of Kabul, especially individuals who do not access formal dental care. Moreover, limited time and resources prevented the collection of more detailed data on other potentially confounding aspects of oral health behavior. The questionnaire was not a fully validated instrument but rather an adaptation of an existing hospital tool, which may limit comparability with studies using standardized measures. Lastly, the findings are relevant to Kabul, which may not be directly generalizable to other regions of Afghanistan.

## 5. Conclusion

This study provides a comprehensive overview of oral hygiene practices and the associated factors among individuals in Kabul, Afghanistan. Our findings revealed that a significant number of participants were exhibiting poor oral hygiene, which requires targeted public health interventions to improve oral health among Afghanistan population. Notably, oral hygiene behaviors were significantly associated with age, education, and wealth status which requires specific consideration while designing oral health interventions and programs. Additionally, the unexpected association between snack consumption and better oral hygiene highlights the importance of considering local dietary habits in oral health strategies. Overall, the findings of this study provide critical guidance for developing contextualized programs including school-based initiatives and community-level interventions in order to promote effective oral hygiene practices in low-resource settings like Afghanistan.

## Figures and Tables

**Table 1:** Description of study participants.

Variables	Measurement		Missing values
	Frequency	Percentage	
Sex			
Male	832	43.1	15
Female	1101	56.9	
Total	1933	100	
Participants age group (mean age: 28.6)			
Under 18	233	12.2	38
18–24	852	44.6	
25–34	426	22.3	
35–44	183	9.8	
45–54	127	6.6	
55–64	57	2.9	
>64	32	1.7	
Total	1910	100	
Education level			
No education	334	17.1	4
Secondary education	277	14.2	
High school	869	44.7	
Higher education	464	23.8	
Total	1944	100	
Wealth status			
Poor	259	13.4	18
Middle	1230	63.7	
Good	441	22.8	
Total	1930	100	
Beverage consumption			
No	374	19.3	18
Sometimes	1219	63.1	
Usually	337	17.4	
Total	1930	100	
Sweet and candy consumption			
No	302	15.5	5
Sometimes	1129	58.1	
Usually	512	26.3	
Total	1943	100	
Citric fruits and foods			
No	380	19.6	9
Sometimes	1086	56.0	
Usually	473	24.3	
Total	1939	100	
Snack			
No	761	39.2	10
Sometimes	865	44.6	
Usually	312	16.1	
Total	1938	100	
Gastric diseases			
No	1335	68.7	4
Yes	609	31.3	
Total	1944	100	
Xerostomia			
No	1551	79.7	3
Yes	394	20.3	
Total	1945	100	
Bruxism			
No	1569	81.1	12
Yes	367	18.9	
Total	1936	100	
Continuous medication use			
No	1651	85.2	9
Yes	288	14.8	
Total	1939	100	
Using dental floss			
No	942	48.5	5
Sometimes	774	39.8	
Usually	227	11.7	
Total	1943	100	
Tooth brushing			
Never	193	9.9	8
After meals	375	19.3	
Mornings	573	29.5	
Nights	617	31.8	
Morning and night	182	9.4	
Total	1940	100	
Dental check-up			
No	675	34.8	8
Every 6 months	150	7.7	
Yearly	128	6.6	
When it is painful	985	50.8	
Total	1940	100	

**Table 2:** Distribution of oral hygiene practice across different demographics and behavior categories.

Variables	Oral hygiene status				Total	
	Poor hygiene		Good hygiene			
	*N*	%	*N*	%	*N*	%
Total	1163	59.7	785	40.3	1948	100%
Sex						
Male	508	43.9	324	41.7	832	43.1
Female	648	56.1	453	58.3	1101	56.9
Total	1156	100	777	100	1933	100
Age categories						
Below 45	993	86.1	701	92.6	1694	88.7
45 Or above	160	13.9	56	7.4	216	11.3
Total	1153	100	757	100	1910	100
Wealth status						
Poor	183	15.9	76	9.8	259	13.4
Middle	762	66.1	468	60.2	1230	63.7
Good	208	18.4	233	29.9	441	22.9
Total	1153	100	777	100	1930	100
Education						
Illiterate	266	22.9	68	8.7	334	17.2
Secondary education	181	15.6	96	12.2	277	14.2
High school	473	40.8	396	50.5	869	44.7
Higher education	240	20.7	224	28.6	464	23.9
Total	1160	100	784	100	1944	100
Beverage consumption						
No	233	20.1	141	18.3	374	19.4
Sometimes	762	65.9	457	59.1	1219	63.2
Usually	162	14.0	175	22.6	337	17.5
Total	1157	100	773	100	1930	100
Sweet and candy						
No	171	14.8	131	16.7	302	15.5
Sometimes	687	59.3	442	56.3	1129	58.1
Usually	300	25.9	212	27.0	512	26.4
Total	1158	100	785	100	1943	100
Citric fruits and food						
No	235	20.3	145	18.5	380	19.6
Sometimes	665	57.5	421	53.8	1086	56.0
Usually	256	22.2	217	27.7	473	24.4
Total	1156	100	783	100	1939	100
Snack						
No	511	44.3	250	31.9	761	39.3
Sometimes	484	41.9	381	48.6	865	44.6
Usually	159	13.8	153	19.5	312	16.1
Total	1154	100	784	100	1938	100
Bruxism						
No	956	82.8	613	78.4	1569	81.1
Yes	198	17.2	169	21.7	367	18.9
Total	1154	100	782	100	1936	100
Xerostomia						
No	934	80.4	617	78.8	1551	79.7
Yes	228	19.6	166	21.2	394	20.3
Total	1162	100	783	100	1945	100
Continuous medication usage						
No	1017	87.9	634	81.7	1651	85.1
Yes	140	12.1	148	18.9	288	14.9
Total	1157	100	782	100	1939	100

**Table 3:** Bivariate and multiple logistic regression.

Variables	COR	95% (CI)	*p*-Value	AOR	95% (CI)	*p*-Value
Sex						
Male	(1) Ref	—	—	—	—	—
Female	1.10	0.91–1.32	0.328	—	—	—
Age group						
Under 45	(1) Ref	—	—	(1) Ref	—	—
Above 45	0.49	0.36–0.68	0.000	0.65	0.45–0.94	0.02
Education level						
Illiterate	(1) Ref	—	—	(1) Ref	—	—
Secondary school	2.07	1.44–2.98	0.000	1.79	1.19–2.68	<0.001
High school	3.27	2.43–4.41	0.000	2.88	2.05–4.05	<0.001
Higher education	3.65	2.64–5.04	0.000	3.01	2.10–4.29	<0.001
Wealth status						
Poor	(1) Ref	—	—	(1) Ref	—	—
Middle	1.47	1.11–1.98	0.008	1.23	0.88–1.72	0.214
Good	2.70	1.95–3.74	0.000	2.29	1.58–3.31	<0.001
Beverage consumption						
No	(1) Ref	—	—	—	—	—
Sometimes	0.99	0.78–1.25	0.941	—	—	—
Usually	1.78	1.32–2.04	0.000	—	—	—
Sweet and candy consumption						
No	(1) Ref	—	—	—	—	—
Sometimes	0.83	0.64–0.108	0.183	—	—	—
Usually	0.92	0.69–1.22	0.582	—	—	—
Citric fruits and food						
No	(1) Ref	—	—	—	—	—
Sometimes	1.02	0.80–1.30	0.834	—	—	—
Usually	1.37	1.04–1.80	0.024	—	—	—
Snack						
No	(1) Ref	—	—	(1) Ref	—	—
Sometimes	1.61	1.31–1.97	0.000	1.34	1.08–1.67	0.007
Usually	1.97	1.51–2.57	0.000	1.66	1.24–2.21	<0.001
Continuous medication use						
No	(1) Ref	—	—	(1) Ref	—	—
Yes	1.70	1.32–2.18	0.000	2.14	1.61–2.83	<0.001

Abbreviations: AOR, adjusted odds ratio; CI, confidence interval; COR, crude odds ratio.

## Data Availability

The data that support the findings of this study are available from the corresponding author upon reasonable request.
